# Supplementary material for: A giant amphipathic helix from a perilipin that is adapted for coating lipid droplets
Source: Nat Commun. 2018 Apr 6;9:1332. doi: 10.1038/s41467-018-03717-8 (PMC5889406; doi:10.1038/s41467-018-03717-8)
Supplement: Supplementary file 1 — Supplementary Information [file 41467_2018_3717_MOESM1_ESM.pdf]

SUPPLEMENTARY INFORMATION

A giant amphipathic helix from a perilipin that is adapted for coating lipid droplets

Alenka Čopič[1]*, Sandra Antoine-Bally[1‡], Manuel Giménez-Andrés[1,2‡], César La Torre Garay[1‡], Bruno Antonny[3], Marco M. Manni[3], Sophie Pagnotta[3], Jeanne Guihot[1], Catherine L. Jackson[1]

[1]Institut Jacques Monod, CNRS, UMR 7592, Université Paris Diderot, Sorbonne Paris Cité, 75013 Paris, France. [2] Université Paris-Sud, Université Paris-Saclay, 91405, Orsay, France. [3] Université Côte d'Azur, CNRS, IPMC, 06560 Valbonne, France.

[‡]*These authors contributed equally to this work.*

*Corresponding Author: Alenka Copic, Institut Jacques Monod, CNRS, UMR 7592, Université Paris Diderot, Sorbonne Paris Cité, 15 rue Hélène Brion 75013 Paris, France.
Phone : +33 1 57 27 80 05
E-mail: alenka.copic@ijm.fr

File contains:

- **Supplementary Figures 1-7**
- **Supplementary Note 1**
- **Supplementary Tables 1-4**
- **Supplementary References**

Supplementary Figure 1a

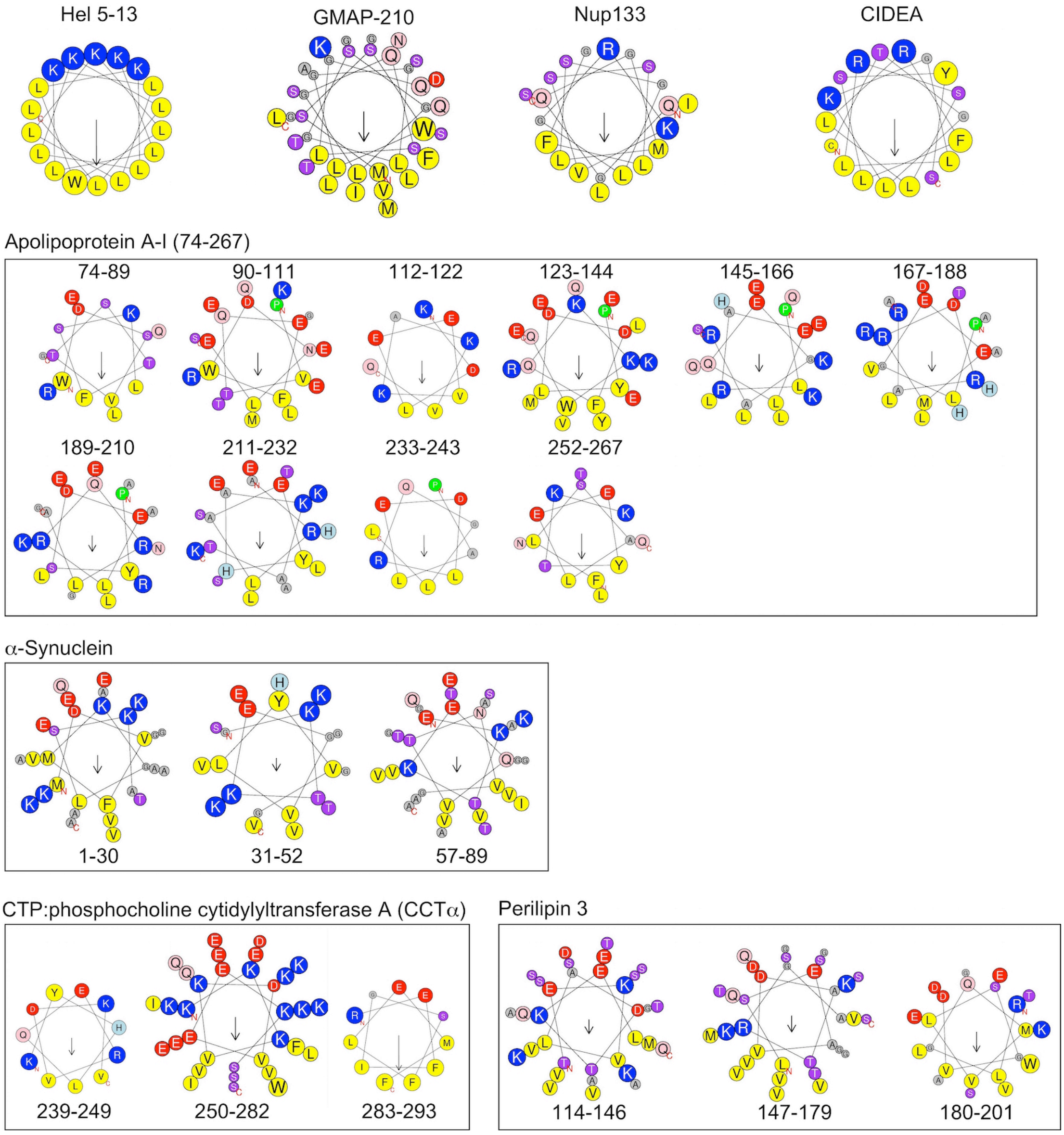

Supplementary Figure 1. Comparison of amphipathic helices. (a) Helical wheel representations of AHs from human proteins described in Table 1. CIDEA, CCTα and perilipin 3 are LD proteins. **(b)** Helical wheel representations of the complete predicted AH sequence of human perilipin 4. AHs are plotted as either classical (5-18) α-helices or as 3-11 helices in the case of sequences containing 11-mer repeats (apolipoprotein A1, α-synuclein, CCTα, perilipin 3, perilipin 4). In the case of longer helices (>36 amino acids), sequences are plotted as consecutive AHs for technical reasons, except in the case of apolipoprotein A1, where proline residues separate consecutive helices as shown[1]. Arrows represent hydrophobic moment. All AHs were plotted using Heliquest[2].

Supplementary Figure 1b
human Plin4AH (aa70-1037)

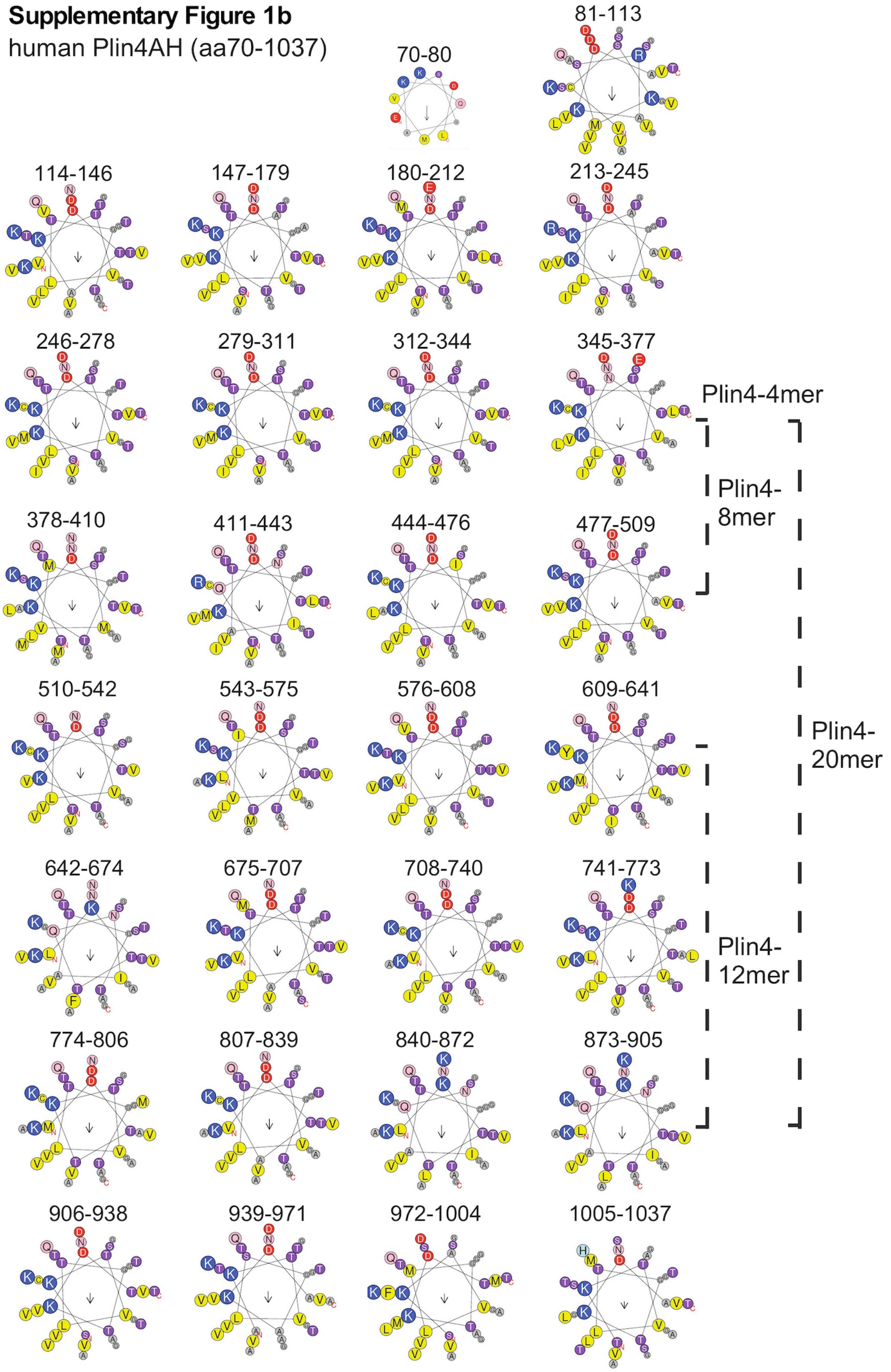

Supplementary Figure 2

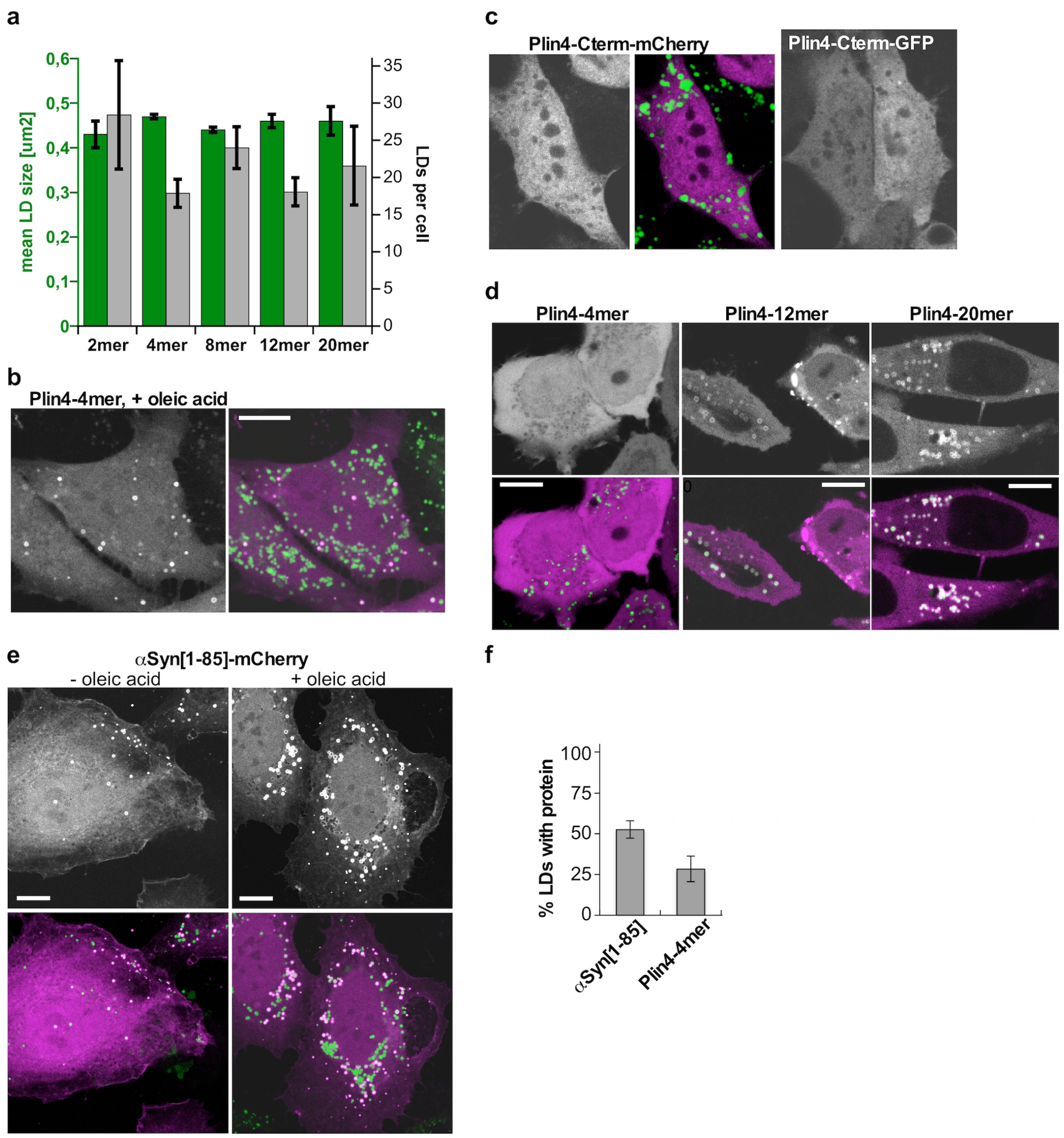

Supplementary Figure 2. Localization of Plin4 and α-synuclein fragments to LDs in HeLa cells. (**a**) Expression of Plin4-AH constructs in HeLa cells does not affect lipid droplet size or number. 40-60 cells from 2 independent experiments were analyzed for each construct (same cells as in Fig. 1f). Error bars show the range of mean values for the 2 experiments. (**b**) Plin4-4mer localizes to a fraction of LDs in fixed HeLa cells when LDs are induced overnight with 250 μM oleic acid. Left panel shows Plin4-4mer, right panel show colocalization with LDs stained with Bodipy. (**c**) The carboxyl-terminal region of Plin4, comprising the predicted 4-helix bundle, does not confer localization to LDs or to other organelles. (**d**) In live cells, localization of Plin4-AH to LDs improves with AH length. Upper panels show localization of Plin4-AH-mCherry constructs (4mer, 12mer and 20mer) in live HeLa cells. Lower panels show colocalization of Plin4-AH (magenta) and LDs stained with Bodipy. Note that Plin4-4mer appears completely cytosolic, whereas Plin4-12mer and Plin4-20mer can be visualized on all LDs, with Plin4-20mer displaying a stronger LD signal. (**e**) The AH of α-synuclein localizes to a fraction of LDs in fixed HeLa cells. The first 85 amino acids of α-synuclein, fused to mCherry (αSyn[1-85]), were transiently expressed in HeLa cells grown overnight in standard medium (upper panel, -OA) or with the addition of 250 mM oleic acid (lower panel, +OA). Fixed cells were imaged using a confocal microscope. LDs are stained with Bodipy. (**f**) Quantification of the fraction of LDs stained with αSyn[1-85] in cells without oleic acid. 21-26 cells from two independent experiments were quantified. Plin4-4mer localization was quantified in 4 independent experiments, 20-37 cells per experiment. Error bar shows the range of means between experiments.

Supplementary Figure 3

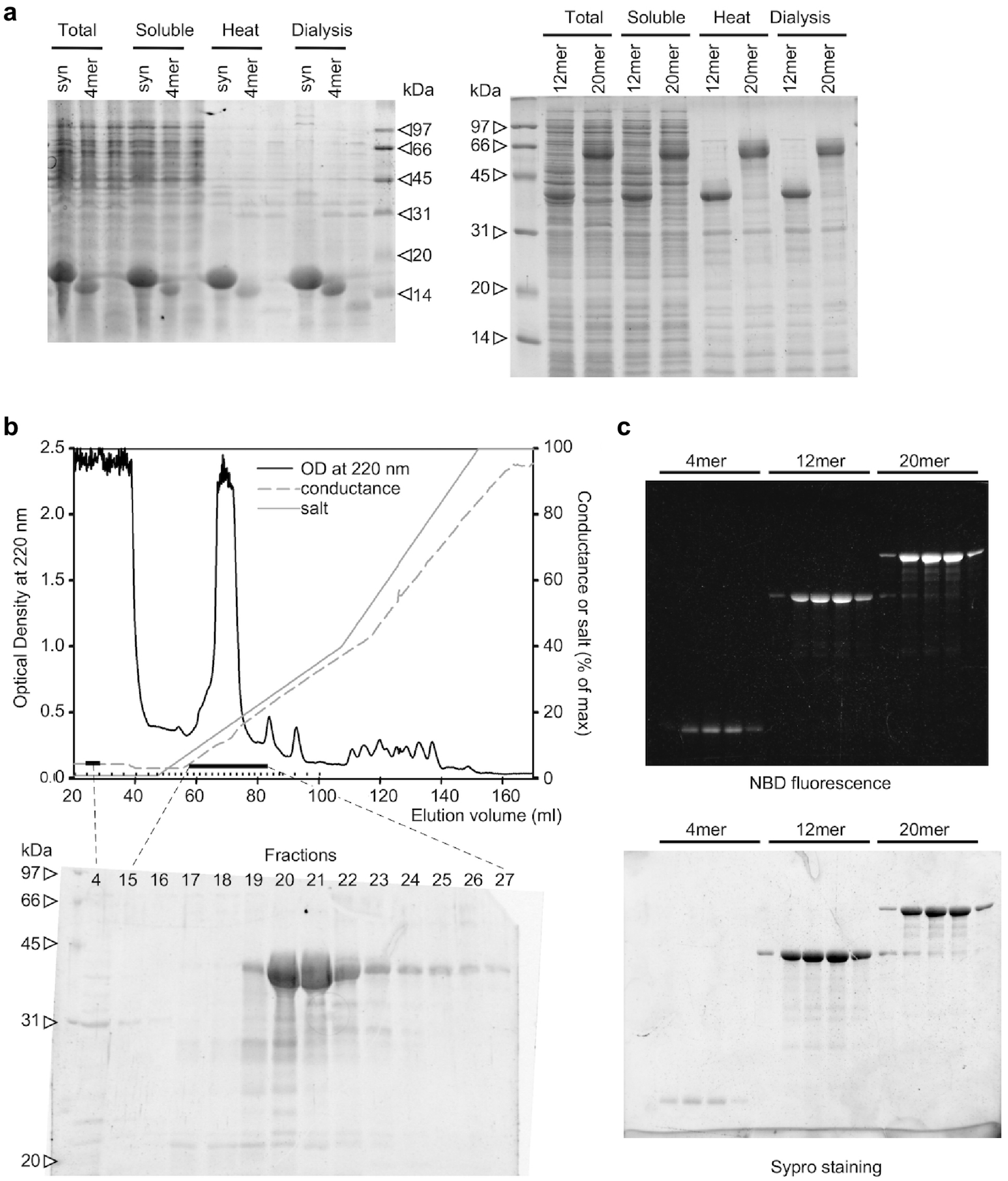

Supplementary Figure 3. Plin4 AH purification and labelling. (a) SDS-PAGE analysis of the indicated Plin4 constructs after expression in E coli. The indicated lanes show the proteins present in the total bacteria extract (total) and after a three step protocol, which includes centrifugation to get the soluble fraction (Soluble), boiling and centrifugation to get the heat-resistant fraction (Heat), and dialysis of the heat resistant fraction against the buffer used for subsequent chromatography (dialysis). This procedure was performed on bacteria expressing Plin4-4mer or alpha-synuclein (upper gel) as well as on bacteria expressing Plin4-12mer or Plin4-20mer (lower gel). Proteins were visualised by Sypro Orange staining. (**b)** Cation exchange chromatography. The example shows the elution of Plin4-12mer from a fraction similar to that shown in **a** on a Source S column submitted to a NaCl gradient from 1 mM to 1000 mM. The continuous grey line shows the theoretical salt gradient, the dashed grey line shows the conductivity, and the continuous black line shows the optical density at 220 nm. The indicated fractions were analysed by SDS-PAGE using Sypro Orange staining. (**c)** NBD labelling. Plin4 4mer, 12mer and 20mer were purified by cation exchange chromatography as in **b,** and then were incubated with an excess of NBD-iodoacetamide. After quenching with DTT, the samples were loaded on a NAP-10 column to separate the proteins from the excess probe. The gel shows the first 5 fractions of the NAP-10 column corresponding to the protein fractions. The gel was directly visualized in a fluorescence imaging system to detect NBD-labeled proteins and then stained with Sypro Orange to detect all proteins.

Supplementary Figure 4

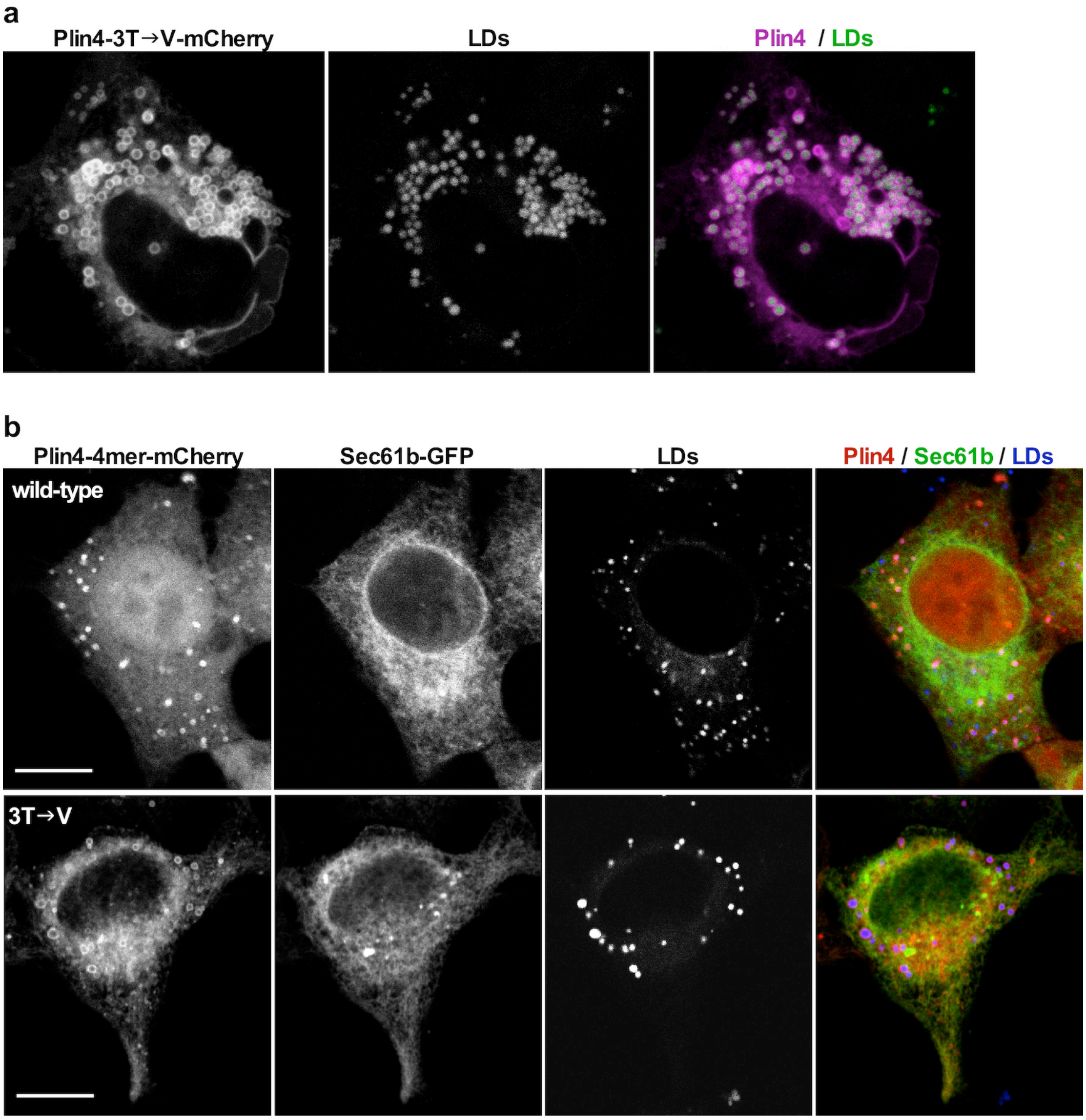

Supplementary Figure 4. Localization of a hydrophobic Plin4 AH mutant in HeLa cells. (a) Localization of the 3T→V Plin4-4mer mutant in live HeLa cells is similar as in fixed cells (see Fig. 3b). (**b)** Wild-type Plin4-4mer-mCherry does not colocalize with the ER marker Sec61b-GFP (transient expression), whereas the more hydrophobic 3T→V mutant shows colocalization, although it probably also localizes to other internal membranes. LDs were stained with Bodipy and cells were fixed before viewing with confocal microscope. Scale bar: 10 µm.

Supplementary Figure 5

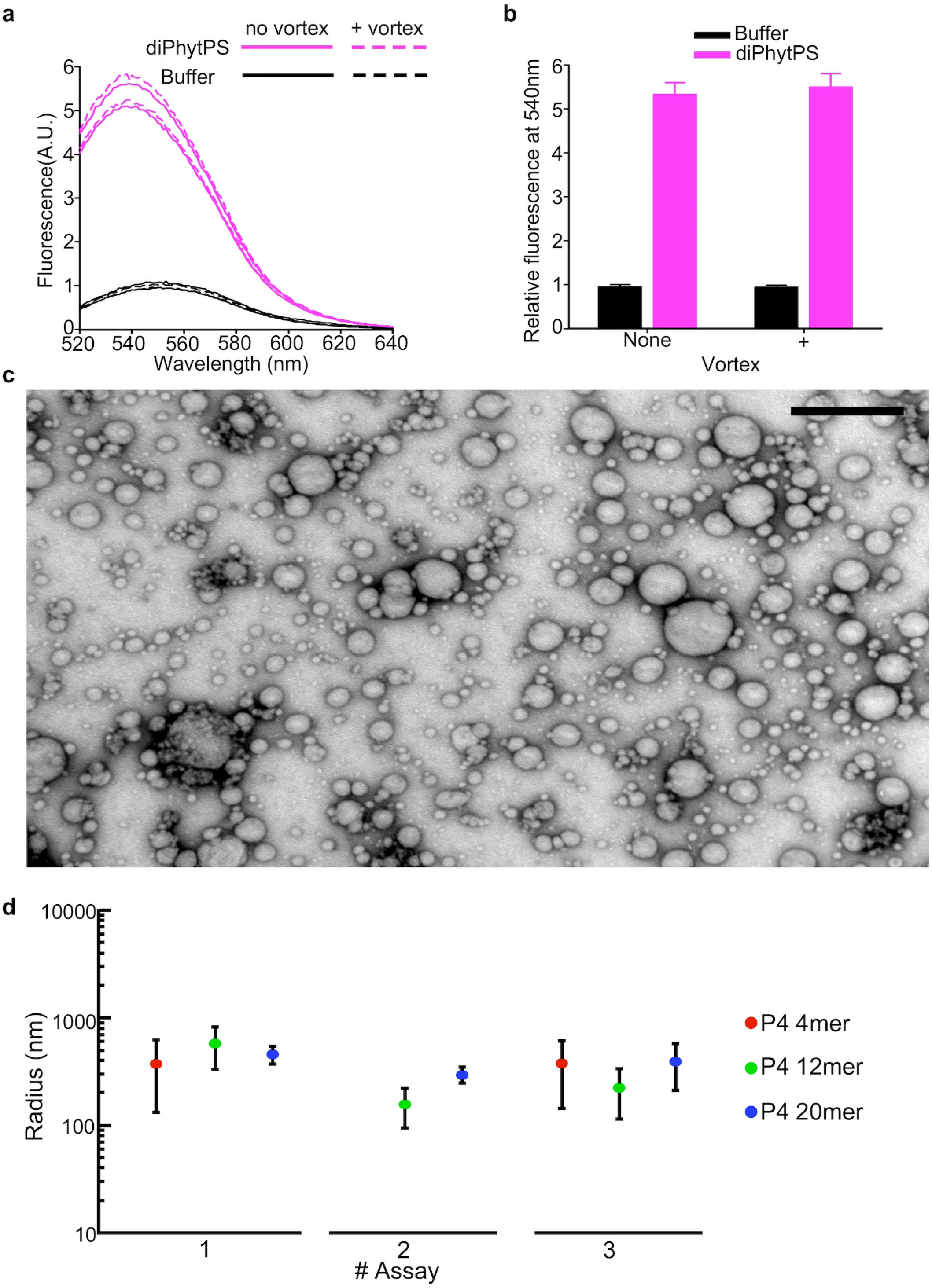

Supplementary Figure 5. Supporting data for experiments with Plin4-oil emulsion. (**a,b**) Vortexing does not affect the liposome binding properties of Plin4 AH. **a**) Fluorescent spectra of NBD-Plin4-4mer before or after 90 s of vigorous vortexing in buffer alone or in buffer containing diphytanoyl-PS liposomes. Fluorescence maxima are plotted in **b**, with error bars showing the variation between two independent experiments. **(c)** Enlarged area of the negative staining electron microscopy image of the Plin4-12mer-triolein emulsion shown in Fig. 6c. Scale bar: 0.5 µm. (**d**) Dynamic light scattering measurement of the size distribution of an aliquot withdrawn from the middle of the oil emulsion obtained with Plin4-4mer, Plin4-12mer, or Plin4-20mer (all at 0.5 mg ml^{-1}) from 3 independent experiments, with dots representing peak maxima and vertical bars representing polydispersity.

Supplementary Figure 6

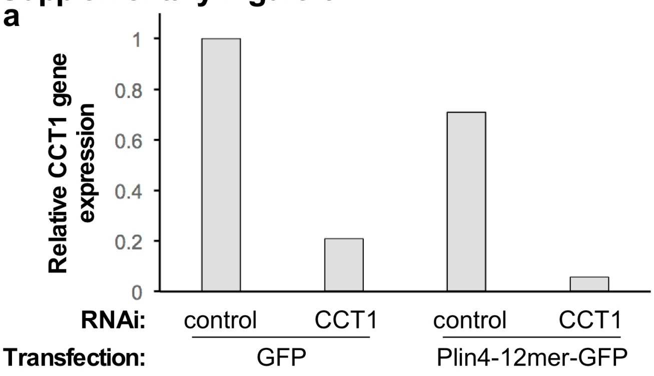

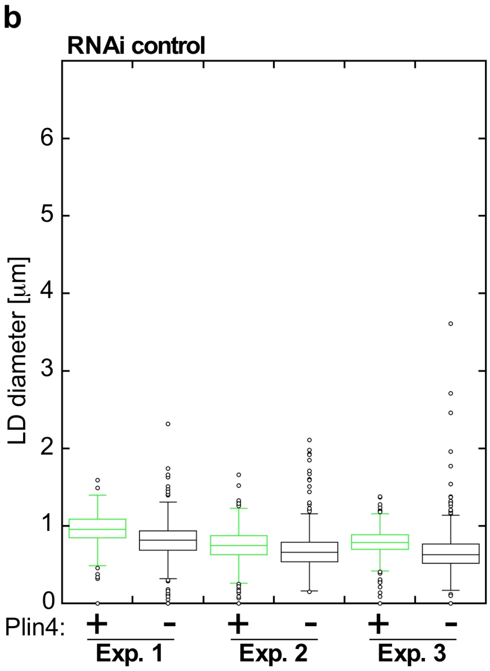

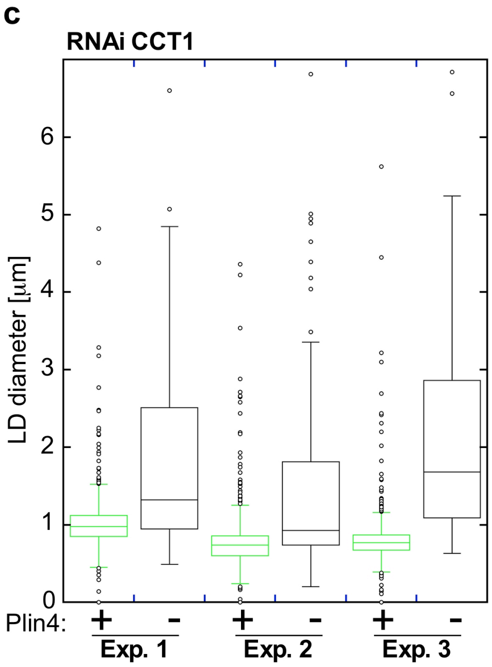

Supplementary Figure 6. CCT1 depletion in Drosophila S2 cells transfected with Plin4-12mer. (a) CCT1 expression was reduced at least five-fold by RNAi treatment of S2 cells that were either stably transfected with Plin4-12mer-GFP or with GFP only. Primers used were the same as in[3], where similar results were obtained. Mean values for quantitative real-time PCR, performed in triplicates, from a representative experiment are shown. (**b, c)** Summary of LD size measurements from three independent RNAi control (**b**) and RNAi CCT1 experiments (**c**), comparing LDs in cells transfected with Plin4-12mer-GFP or in non-transfected cells. Each box-and-whisker plot represents LDs from 10 cells, with horizontal bars showing the median, boxes showing first and third quartiles, and vertical bars showing range of values. Data were analyzed using Wilcoxon Mann–Whitney test for unpaired data.

Supplementary Figure 7. Images of uncropped gels and Western blot membranes.

Uncropped gel for Figure 6f

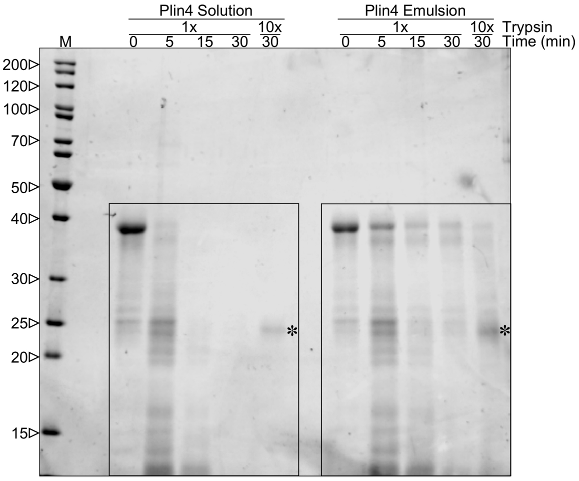

Uncropped gel for Figure 6g,h

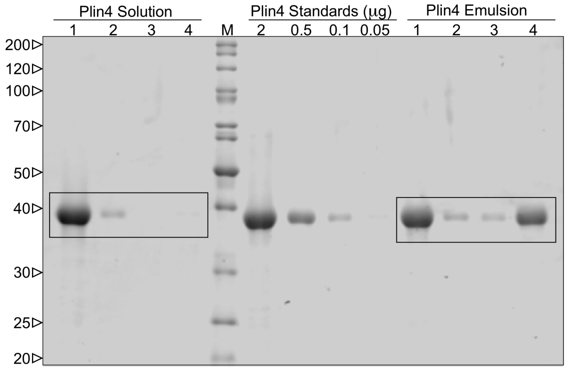

Uncropped Western blot membranes for Figure 7b

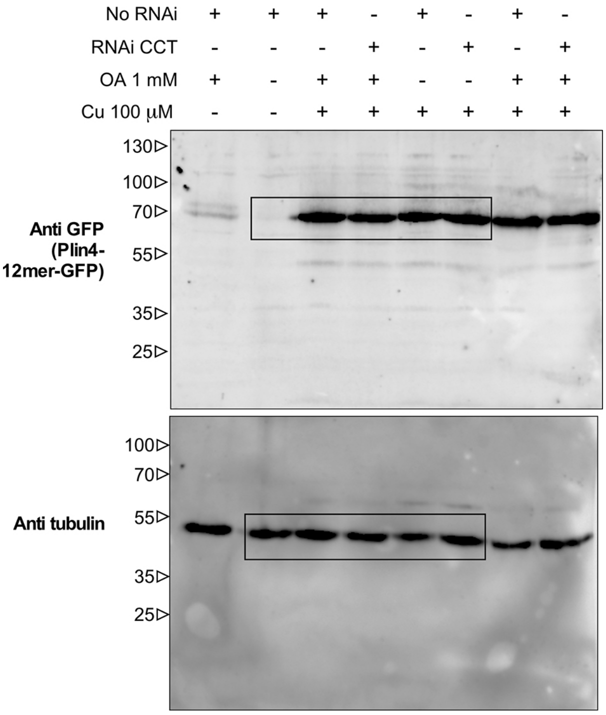

Supplementary Figure 7. Images of uncropped gels and Western blot membranes.

Supplementary Note 1. Coverage of triolein by Plin4-12mer in emulsion experiments

We estimate the efficiency of oil coverage by Plin4 from the yield of *in vitro* emulsion experiments (see Fig. 6).

In these experiments, about 10 nmoles of Plin4 12mer convert 10 µl of triolein into small droplets with a mean radius $R_{drop} \approx 200$ nm.

The number of droplets N_{drop} is given by the ratio between the volume of oil (V_{oil}) and the droplet volume

$V_{oil} = 10\ \mu l = 10^{19}\ nm^3$

$V_{drop} = (4/3)\pi R_{drop}^3 = 3.3 \times 10^7\ nm^3$

$N_{drop} = V_{oil}/V_{drop} = 3 \times 10^{11}$

The surface of each droplet is

$s_{drop} = 4\pi R_{drop}^2 \approx 5 \times 10^5\ nm^2$.

Consequently, the total surface of oil exposed to buffer is

$S_{oil} = N_{drop} \times s_{drop} = 1.5 \times 10^{17}\ nm^2$

This surface should then be compared to the area of Plin4 molecules in a helical conformation.

Assuming that Plin4 adopts a perfectly helical conformation when in contact with neutral lipids, each Plin4 33-mer repeat covers an area:

A_{1mer} = 1 nm (helix width) x 0.15 nm (length/amino-acid) x 33 (number of amino-acid) ≈ 5 nm^2.

The value of 1 nm corresponds to the width of the apolipoprotein helix when forming a minimal discoidal lipid particle [4]. In such a particle, two anti-parallel helices are in tight contact with each other.

The area covered by Plin4 12mer, which was used in the experiment of Fig. 6, is

$A_{12mer} = 5 \times 12 = 60\ nm^2$

After flotation, 10% of Plin4 (= 1 nmole or 10^{-9} mole) is recovered in the floating fraction and thus coats the droplets (Fig. 6g). The total area of Plin4 12mer helix is

$A_{Protein} = 10^{-9} \times N_{Av} \times A_{12mer} = 10^{-9} \times 6.02 \times 10^{23} \times 60 = 3.6 \times 10^{16}\ nm^2$ where N_{Av} is the Avogadro number.

These calculations indicate that the total surface of protein ($A_{Protein}$) is in the same range as the total surface of droplets (S_{oil}). However, the exact values given by these estimations should be taken with caution. First, the oil surface is difficult to assess because the oil droplets are not homogeneous in size (see Fig. 6b,c). We can see both large drops of oil (a few µm in diameter) in the fluorescent-labeled emulsion (Fig. 6d) as well as very small droplets (≈ 10 nm in diameter) by EM (Fig. 6c). In addition, we have not been able to measure the amount of residual oil that has not been incorporated into the protein-oil emulsion. Second, the exact amount of Plin4 is difficult to determine because the amino acid composition of this protein makes it poorly adapted to classical protein concentration assays (see Methods). Third, the exact elementary surface covered by each Plin4 33-mer repeat cannot be precisely known without knowledge of its exact structure. Notwithstanding these limitations, the good match between the two surfaces makes a mechanism of oil coverage by a coat consisting of packed Plin4 molecules a plausible model.

Supplementary Table 1.

Comparison of Plin4 AH amino acid composition with protein averages.

Amino acid	Plin4 AH [mol%] (1)	α-helix average [mol%] (2)	vertebrate average [mol%] (2)	Plin4 AH / vert. average
Ala	10.7	7.05	7.4	1.45
Arg	0.4	6.05	4.2	0.10
Asn	3.3	3.8	4.4	0.75
Asp	5.3	4.95	5.9	0.90
Cys	1.2	3.3	3.3	0.36
Gln	3.4	6.35	5.8	0.59
Glu	0.4	7.95	3.7	0.11
Gly	15.4	2.15	7.4	2.08
His	0.1	5.25	2.9	0.03
Ile	1.3	5.45	3.8	0.34
Leu	5.4	6.7	7.6	0.71
Lys	8.8	6.15	7.2	1.22
Met	2.3	6.5	1.8	1.28
Phe	0.3	5.8	4	0.08
Pro	0	1.7	5	0.00
Ser	5.5	2.85	8.1	0.68
Thr	20.9	3.8	6.2	3.37
Trp	0.1	5.1	1.3	0.08
Tyr	0.1	3.7	3.3	0.03
Val	14.9	4.5	6.8	2.19
Negative	9.2			
Positive	5.7			

(1) Composition of amino acid region 67-1042 from human Plin4.
(2) Data from[5].
Color code legend: amino acids that are under-represented compared to vertebrate average are highlighted in light blue (less) or in blue (more), and over-represented amino acids are highlghted in light orange (less) or in orange (more).

Supplementary Table 2. Summary of Plin4 AH mutants used in this study.

**Construct**	**Description**	**Length [aa]**	**<H> (1)**	**<µH> (2)**	**net charge**
Plin4-4mer	WT S246-T377 (3)	132	0.293	0.257	+1
1T→V	[T8V] (4)	132	0.322	0.283	+1
2T→V	[T8V, T26V]	132	0.351	0.299	+1
3T→V	[T8V, T26V, T33V]	132	0.38	0.302	+1
3T→A	[T8A, T26A, T33A]	132	0.297	0.259	+1
4T→S	[T8S, T11S, T26S, T33S]	132	0.256	0.248	+1
2K→Q	[K9Q, K24Q]	132	0.339	0.264	-1
2K→Q, 2T→V	[K9Q, K24Q, T8V, T26V]	132	0.398	0.299	-1
2D→N	[D10N, D32N]	132	0.303	0.247	+3
2D→N, 2T→V	[D10N, D32N, T8V, T26V]	132	0.361	0.288	+3
charge swap, csw	["KCK/KGT, DND/TSG"]	132	0.293	0.291	+1
csw, 2T→V	(5)	132	0.351	0.315	+1

(1) Hydrophobicity of one 33-mer repeat was calculated with Heliquest[2].
(2) Hydrophobic moment of one 33-mer repeat was calculated with Heliquest.
(3) This is wild-type sequence from human Plin4, comprising amino acids 246-377 (4 33mer repeats). Note that the first 3 repeats have the same sequence.
(4) All 4mer mutants are based on a single 33mer repeat from the wild-type sequence (aa246-278), repeated 4 times. Position and substitutions in the 33mer repeat are indicated.
(5) This mutant contains all charge-swap mutations, with additional [T8V, T26V] substitution.

Supplementary Table 3. Plasmids used in this study.

Name	Insert	Region (aa) / length (1)	Vector	Host (2)	Source
pCLG28	Plin4-2mer	hPlin4 246-309 / 66	pmCherry-N1	Mamm	This study
pACJ22	Plin4-4mer	hPlin4 246-377 / 132	pmCherry-N1	Mamm	This study
pCLG26	Plin4-8mer	hPlin4 246-509 / 264	pmCherry-N1	Mamm	This study
pCLG62	Plin4-12mer	hPlin4 510-905 / 396	pmCherry-N1	Mamm	This study
pCLG70	Plin4-20mer	hPlin4 246-905 / 660	pmCherry-N1	Mamm	This study
pKTD1-16A	mouse Plin4	1-1356 (mouse full length)	pcDNA	Mamm	Ref[6]
pCLG47	Plin4-Cterm	1060-1356 (mouse) / 296	pmCherry-N1	Mamm	This study
pCLG48	Plin4-Cterm	1060-1356 (mouse) / 296	pEGFP-N1	Mamm	This study
pCLG34	P4-1T→V	4x[246-278 M8t](3) / 132	pmCherry-N1	Mamm	This study
pCLG36	P4-2T→V	4x[246-278 M10t2] / 132	pmCherry-N1	Mamm	This study
pACJ27	P4-3T→V	4x[246-278 M4t] / 132	pmCherry-N1	Mamm	This study
pACJ43	P4-3T→A	4x[246-278 M9ta] / 132	pmCherry-N1	Mamm	This study
pSB49	P4-4T→S	4x[246-278 M5t] / 132	pmCherry-N1	Mamm	This study
pCLG38	P4-2K→Q	4x[246-278 M11n] / 132	pmCherry-N1	Mamm	This study
pCLG40	P4-2K→Q,2T→V	4x[246-278 M12nt] / 132	pmCherry-N1	Mamm	This study
pSB22	P4-2D→N	4x[246-278 M15d] / 132	pmCherry-N1	Mamm	This study
pSB24	P4-2D→N,2T→V	4x[246-278 M16dt] / 132	pmCherry-N1	Mamm	This study
pACJ33	P4-charge swap	4x[246-278 M3kd] / 132	pmCherry-N1	Mamm	This study
pACJ41	P4-charge swap,2T→V	4x[246-278 M7kt] / 132	pmCherry-N1	Mamm	This study
pCLG05	α-synuclein[1-85]	[1 - 85] / 85	pmCherry-N1	Mamm	This study
pSec61β	Sec61β (AddGene #15108)		pAcGFP-C1	Mamm	T. Rapoport
pRHT140	GFP(C-term fusion)		pRS416-ADHpr	Yeast	S. Leon
pKE28	NheI for subcloning		pRHT140	Yeast	This study
pKE31	PLin4-4mer	hPlin4 246-377 / 132	pKE28	Yeast	This study
pKE33	Plin4-12mer	hPlin4 510-905 / 396	pKE28	Yeast	This study
pKE35	P4-2T→V	4x[246-278 M10t2] / 132	pKE28	Yeast	This study
pKE41	P4-2K→Q,2T→V	4x[246-278 M12nt] / 132	pKE28	Yeast	This study
pCLG03	PLin4-4mer	hPlin4 246-377 / 132	pET21b	E. coli	This study
pKE23	Plin4-12mer	hPlin4 510-905 / 396	pET21b	E. coli	This study
pKE25	Plin4-20mer	hPlin4 246-905 / 660	pET21b-3stop	E. coli	This study
pKE44	P4-2T→V	4x[246-278 M10t2] / 132	pET21b	E. coli	This study
pMTWG	Puro-MTpr—GFP		pAWG	Dros.	This study
pSB41	Plin4-12mer	hPlin4 510-905 / 396	pMTWG	Dros.	This study

(1) Position amino acids in the human Plin4 primary sequence is given, unless otherwise noted.
(2) Mamm: mammalian cells, Dros.: Drosophila cells
(3) All mutants are 4 repeats of the same amino acid sequence; see Supplementary Table 4.

Supplementary Table 4. Synthetic genes used in this study.

Name	Construct	protein sequence (full length)	DNA sequence
PLin4-4mer	Plin4[246-377]	MSKTVLTGTKDTVCSGVTGAMNVAKGTIQTGVDTSKTVLTGTKDTVCSGVTGAMNVAKGTIQTGVDTSKTVLTGTKDTVCSGVTGAMNVAKGTIQTGVDTtKTVLTGTKnTVCSGVTGAvNlAKeaIQgGlDT	**CTCGAG***atg* AGCAAAACCGTGCTCACTGGGACGAAGGATACCGTGTGTTCTGGCGTAACCGGTGCCATGAACGTGGCAAAAGGCACCATTCAGACAGGGGTGGATACCAGCAAGACTGTCCTGACAGGCACCAAGGACACGGTTTGTTCCGGTGTCACAGGAGCGATGAATGTGGCCAAAGGTACCATCCAGACTGGCGTAGACACTTCCAAGACAGTGCTGACAGGAACTAAGGACACTGTCTGCTCAGGCGTTACAGGGGCTATGAACGTGGCTAAGGGCACCATCCAGACAGGCGTTGACACAACCAAAACCGTGCTTACGGGGACTAAGAACACTGTCTGCAGTGGAGTGACAGGAGCAGTCAATCTGGCCAAAGAGGCCATACAAGGAGGGTTGGATACC*aa* **GGATCC**
Plin4-8mer	Plin4[246-509]	MSKTVLTGTKDTVCSGVTGAMNVAKGTIQTGVDTSKTVLTGTKDTVCSGVTGAMNVAKGTIQTGVDTSKTVLTGTKDTVCSGVTGAMNVAKGTIQTGVDTTKTVLTGTKNTVCSGVTGAVNLAKEAIQGGLDTTKSMVMGTKDTMSTGLTGAANVAKGAMQTGLNTTQNIATGTKDTVCSGVTGAMNLARGTIQTGVDTTKIVLTGTKDTVCSGVTGAANVAKGAVQGGLDTTKSVLTGTKDAVSTGLTGAVNVAKGTVQTGVDTKD	ctcgagatgTCCAAGACAGTCCTCACCGGCACTAAAGACACAGTGTGTTCTGGCGTTACCGGCGCCATGAATGTAGCCAAAGGAACAATCCAAACTGGTGTGGACACCTCCAAGACCGTCTTGACAGGAACCAAGGATACTGTCTGTAGCGGAGTGACTGGCGCGATGAATGTGGCAAAAGGCACCATACAGACCGGGGTTGATACATCCAAGACAGTGCTCACCGGGACAAAGGATACGGTGTGTAGTGGTGTCACAGGGGCTATGAACGTGGCGAAGGGCACGATTCAGACTGGGGTCGACACCACGAAAACAGTCCTGACTGGCACAAAGAACACCGTTTGCTCAGGTGTGACAGGCGCAGTGAACCTTGCCAAGGAGGCCATTCAGGGTGGGTTGGATACCACTAAAAGCATGGTTATGGGGACCAAGGATACCATGAGCACAGGACTGACTGGCGCTGCAAACGTGGCAAAAGGCGCCATGCAGACAGGCCTGAATACGACTCAGAACATTGCGACCGGTACAAAAGACACTGTTTGCTCTGGGGTGACCGGTGCCATGAATCTGGCTAGAGGAACGATCCAGACAGGCGTAGACACTACTAAGATCGTGCTGACCGGAACTAAGGATACCGTGTGCAGCGGTGTTACTGGGGCAGCCAACGTAGCTAAAGGAGCCGTGCAAGGAGGGCTGGATACCACGAAGAGTGTGCTGACCGGCACGAAAGACGCTGTATCAACCGGGCTTACAGGCGCTGTCAATGTCGCCAAAGGAACCGTGCAGACAGGAGTTGACACTaaggatcc
Plin4-12mer	Plin4[510-905]	MTKTVLTGTKDTVCSGVTSAVNVAKGAVQGGLDTTKSVVIGTKDTMSTGLTGAANVAKGAVQTGVDTAKTVLTGTKDTVTTGLVGAVNVAKGTVQTGMDTTKTVLTGTKDTIYSGVTSAVNVAKGAVQTGLKTTQNIATGTKNTFGSGVTSAVNVAKGAAQTGVDTAKTVLTGTKDTVTTGLMGAVNVAKGTVQTSVDTTKTVLTGTKDTVCSGVTGAANVAKGAIQGGLDTTKSVLTGTKDAVSTGLTGAVKLAKGTVQTGMDTTKTVLTGTKDAVCSGVTGAANVAKGAVQMGVDTAKTVLTGTKDTVCSGVTGAANVAKGAVQTGLKTTQNIATGTKNTLGSGVTGAAKVAKGAVQGGLDTTKSVLTGTKDAVSTGLTGAVNLAKGTVQTGVDTKD	**CTCGAG**atgACCAAAACCGTGCTTACCGGCACCAAAGACACGGTTTGCAGTGGCGTTACATCTGCCGTCAATGTTGCCAAGGGTGCAGTGCAAGGTGGTCTGGACACGACCAAAAGCGTGGTAATCGGGACCAAGGATACCATGTCCACAGGACTGACCGGAGCTGCGAATGTCGCTAAGGGAGCAGTGCAGACTGGAGTCGATACCGCTAAGACCGTTCTTACTGGGACGAAAGACACAGTTACCACCGGACTGGTTGGGGCCGTAAACGTAGCCAAGGGGACAGTCCAGACAGGTATGGACACGACCAAGACTGTTCTGACTGGCACCAAGGACACAATCTACTCTGGGGTCACATCAGCTGTGAACGTAGCCAAAGGGGCTGTGCAAACAGGCCTCAAAACCACCCAGAACATTGCCACGGGGACTAAGAATACCTTCGGCTCAGGCGTGACCTCCGCTGTGAATGTGGCCAAGGGAGCAGCTCAGACGGGCGTAGACACAGCCAAGACTGTGCTGACTGGTACTAAGGACACTGTGACCACTGGACTGATGGGAGCGGTGAATGTTGCCAAAGGCACCGTGCAGACTTCCGTGGACACTACTAAGACCGTTCTCACT*GGAACTAAGGACACCGTTTGT* AGCGGGGTGACTGGCGCAGCGAATGTCGCCAAGGGCGCCATTCAGGGCGGCTTGGATACCACAAAATCCGTCTTGACCGGCACAAAAGATGCCGTAAGCACAGGCCTTACCGGCGCCGTGAAACTGGCCAAAGGGACGGTGCAAACTGGCATGGACACGACAAAGACCGTCCTGACCGGGACCAAGGATGCTGTCTGCTCAGGTGTGACCGGCGCTGCAAACGTGGCCAAGGGTGCGGTCCAGATGGGAGTGGATACAGCCAAAACCGTGTTGACAGGGACAAAGGATACTGTCTGTAGCGGTGTTACGGGCGCCGCAAACGTCGCCAAAGGGGCGGTCCAGACAGGACTCAAGACGACCCAGAACATAGCAACAGGGACAAAGAACACCCTCGGTAGTGGAGTGACAGGGGCTGCAAAAGTGGCAAAGGGCGCTGTGCAAGGCGGCCTGGATACTACAAAGTCTGTCCTGACTGGAACTAAAGACGCAGTGAGCACAGGACTGACTGGAGCTGTGAATCTGGCAAAAGGGACAGTACAGACAGGTGTGGATACAaa**ggatcc**
Plin4-1T>V	4xM8t1	SKTVLTGvKDTVCSGVTGAMNVAKGTIQTGVDTSKTVLTGvKDTVCSGVTGAMNVAKGTIQTGVDTSKTVLTGvKDTVCSGVTGAMNVAKGTIQTGVDTSKTVLTGvKDTVCSGVTGAMNVAKGTIQTGVDT	CTCGAGatgTCCAAGACAGTGCTCACAGGCGTCAAAGACACCGTGTGTAGTGGGGTTACTGGGGCTATGAATGTGGCCAAAGGAACCATCCAGACTGGAGTCGATACCTCCAAGACCGTACTGACTGGTGTGAAAGACACCGTCTGCTCAGGGGTTACTGGTGCCATGAATGTCGCGAAAGGCACGATTCAGACAGGTGTGGACACAAGCAAGACAGTGCTGACAGGCGTAAAGGACACTGTATGCTCTGGCGTTACTGGGGCAATGAACGTCGCTAAAGGGACCATACAGACTGGCGTGGATACGAGCAAGACCGTGCTTACCGGAGTCAAGGATACCGTGTGTTCTGGCGTGACAGGCGCAATGAACGTTGCCAAGGGAACCATCCAAACGGGAGTGGATACAaaGGATCC
Plin4-2T>V	4xM10t2	SKTVLTGvKDTVCSGVTGAMNVAKGvIQTGVDTSKTVLTGvKDTVCSGVTGAMNVAKGvIQTGVDTSKTVLTGvKDTVCSGVTGAMNVAKGvIQTGVDTSKTVLTGvKDTVCSGVTGAMNVAKGvIQTGVDT	ctcgagatgAGCAAGACCGTACTGACTGGGGTTAAGGACACCGTCTGTTCTGGCGTTACTGGTGCGATGAATGTCGCCAAAGGCGTGATTCAGACAGGAGTGGATACCTCCAAGACGGTGCTTACAGGCGTGAAAGACACTGTGTGTTCAGGCGTTACAGGCGCAATGAACGTCGCAAAAGGTGTGATCCAAACGGGCGTGGATACCAGCAAGACGGTGCTGACAGGGGTCAAGGATACCGTTTGCTCTGGAGTTACAGGGGCTATGAACGTAGCCAAAGGGGTGATACAGACCGGAGTGGACACTTCCAAGACTGTGCTCACTGGTGTGAAGGACACAGTCTGCAGTGGAGTCACAGGAGCCATGAATGTGGCTAAAGGGGTCATCCAGACCGGCGTAGATACCaaggatcc
Plin4-3T>V	4xM4t	SKTVLTGvKDTVCSGVTGAMNVAKGvIQTGVDvSKTVLTGvKDTVCSGVTGAMNVAKGvIQTGVDvSKTVLTGvKDTVCSGVTGAMNVAKGvIQTGVDvSKTVLTGvKDTVCSGVTGAMNVAKGvIQTGVDv	CTCGAGa**tgTCCAAGACAGTTCTGACC**GGCGTGAAGGACACCGTATGCTCAGGGGTCACTGGTGCCATGAATGTCGCCAAAGGAGTGATTCAGACTGGAGTGGATGTCAGCAAGACAGTGCTTACTGGCGTGAAAGACACGGTGTGTAGTGGAGTTACAGGCGCCATGAACGTGGCAAAGGGAGTGATCCAGACTGGGGTTGACGTGAGCAAGACCGTACTCACAGGCGTCAAGGATACGGTTTGCTCTGGCGTAACTGGCGCTATGAACGTAGCGAAAGGAGTGATCCAAACCGGCGTTGATGTCTCCAAGACCGTTCTGACCGGTGTCAAAGACACCGTCTGTTCTGGGGTGACAGGGGCAATGAATGTGGCTAAAGGGGTGATACAGACAGGTGTGGATGTGaaGGATCC

Plin4-3T>A	4xM9ta	SKTVLTGaKDTVCSGVTGAMNVAKGaIQTGVDaSKTVLTGaKDTVCSGVTGAMNVAKGaIQTGVDaSKTVLTGaKDTVCSGVTGAMNVAKGaIQTGVDaSKTVLTGaKDTVCSGVTGAMNVAKGaIQTGVDa	CTCGAGatgTCCAAGACAGTTCTGACAGGGGCTAAGGATACGGTGTGTTCTGGGGTGACAGGTGCCATGAACGTCGCCAAAGGCGCTATCCAGACAGGGGTTGACGCAAGCAAAACCGTCCTGACTGGCGCAAAAGACACTGTGTGCTCAGGAGTTACCGGTGCCATGAATGTGGCAAAGGGAGCCATCCAAACCGGCGTGGATGCGTCTAAGACAGTCCTTACCGGAGCAAAGGATACCGTGTGTAGCGGGGTAACCGGAGCCATGAATGTCGCCAAGGGCGCCATACAGACTGGCGTTGATGCCTCCAAGACTGTCCTCACTGGCGCTAAAGACACGGTGTGCAGTGGAGTGACAGGCGCAATGAACGTAGCTAAAGGGGCGATTCAGACCGGTGTGGACGCTaaGGATCC
Plin4-4T>S	4xM5t	SKTVLTGSKDSVCSGVTGAMNVAKGSIQTGVDSSKTVLTGSKDSVCSGVTGAMNVAKGSIQTGVDSSKTVLTGSKDSVCSGVTGAMNVAKGSIQTGVDSSKTVLTGSKDSVCSGVTGAMNVAKGSIQTGVDS	AGCAAAACCGTGCTCACTGGGagcAAGGATtccGTGTGTTCTGGCGTAACCGGTGCCATGAACGTGGCAAAAGGCtcaATTCAGACAGGGGTGGATtctAGCAAGACTGTCCTGACAGGCtccAAGGACagcGTTTGTTCCGGTGTCACAGGAGCGATGAATGTGGCCAAAGGTtctATCCAGACTGGCGTAGACagtTCCAAGACAGTGCTGACAGGAagtAAGGACtcaGTCTGCTCAGGCGTTACAGGGGCTATGAACGTGGCTAAGGGCtccATCCAGACAGGCGTTGACtcaagcAAAACCGTGCTTACGGGGtctAAGgacagtGTCTGCAGTGGAGTGACAGGAGCAatgAATgtcGCCAAAggtagcATACAAacgGGGgtaGATtcc
Plin4-2K>Q	4xM11n	SKTVLTGTqDTVCSGVTGAMNVAqGTIQTGVDTSKTVLTGTqDTVCSGVTGAMNVAqGTIQTGVDTSKTVLTGTqDTVCSGVTGAMNVAqGTIQTGVDTSKTVLTGTqDTVCSGVTGAMNVAqGTIQTGVDT	CTCGAGatgAGCAAAACCGTGCTTACCGGTACTCAGGACACTGTGTGCTCTGGCGTGACAGGTGCCATGAATGTGGCACAGGGGACCATACAGACCGGTGTCGATACGTCCAAGACCGTGCTGACTGGCACACAGGACACTGTCTGCAGTGGCGTAACTGGAGCCATGAATGTCGCACAAGGCACCATTCAGACTGGGGTGGATACCTCCAAGACCGTTCTCACGGGAACACAGGATACCGTGTGTTCTGGGGTTACAGGAGCCATGAACGTGGCTCAAGGCACAATCCAGACAGGCGTTGATACGAGCAAAACCGTCCTGACAGGCACGCAAGACACCGTGTGTTCAGGGGTAACTGGAGCGATGAACGTCGCTCAGGGAACAATCCAGACTGGGGTTGACACAaaGGATCC
Plin4-2K>Q,2T>V	4xM12nt	SKTVLTGvqDTVCSGVTGAMNVAqGvIQTGVDTSKTVLTGvqDTVCSGVTGAMNVAqGvIQTGVDTSKTVLTGvqDTVCSGVTGAMNVAqGvIQTGVDTSKTVLTGvqDTVCSGVTGAMNVAqGvIQTGVDT	ctcgagatgTCCAAAACCGTGCTGACTGGAGTCCAGGACACTGTGTGTTCTGGCGTTACTGGCGCGATGAATGTGGCTCAAGGCGTGATACAGACAGGCGTGGATACCAGCAAGACCGTGCTCACTGGGGTGCAAGACACGGTGTGCAGTGGTGTCACTGGAGCCATGAACGTTGCACAAGGGGTGATTCAGACCGGAGTGGATACCTCCAAGACAGTGCTGACAGGCGTACAGGACACTGTCTGCTCAGGCGTAACAGGGGCCATGAACGTCGCTCAGGGTGTCATCCAGACGGGTGTCGATACGAGCAAAACCGTGCTTACCGGGGTTCAGGATACCGTGTGTTCTGGGGTAACAGGAGCCATGAATGTTGCACAGGGCGTTATCCAGACAGGAGTCGACACAaaggatcc
Plin4-2D>N	P4-M15d	SKTVLTGTKNTVCSGVTGAMNVAKGTIQTGVNTSKTVLTGTKNTVCSGVTGAMNVAKGTIQTGVNTSKTVLTGTKNTVCSGVTGAMNVAKGTIQTGVNTSKTVLTGTKNTVCSGVTGAMNVAKGTIQTGVNT	AGCAAAACGGTACTCACTGGCACAAAGAACACGGTTTGCAGTGGAGTCACTGGAGCCATGAATGTGGCAAAAGGCACCATTCAGACTGGGGTGAATACCTCCAAAACCGTCCTGACAGGAACCAAGAACACAGTGTGCTCTGGAGTCACTGGGGCAATGAATGTTGCCAAGGGTACCATCCAAACTGGGGTTAACACCTCCAAGACTGTCCTTACAGGCACGAAGAATACCGTCTGTTCTGGTGTAACCGGTGCTATGAATGTGGCGAAAGGGACGATACAGACAGGCGTGAACACAAGCAAGACAGTGCTGACTGGCACCAAGAACACCGTGTGTTCAGGCGTTACAGGCGCTATGAACGTGGCCAAAGGAACTATCCAGACAGGGGTGAATACC
Plin4-2D>N,2T>V	P4-M16dt	SKTVLTGVKNTVCSGVTGAMNVAKGVIQTGVNTSKTVLTGVKNTVCSGVTGAMNVAKGVIQTGVNTSKTVLTGVKNTVCSGVTGAMNVAKGVIQTGVNTSKTVLTGVKNTVCSGVTGAMNVAKGVIQTGVNTKD	CTCGAGatgAGCAAAACCGTCCTTACAGGCGTGAAGAACACAGTATGCTCAGGAGTGACTGGAGCCATGAATGTGGCAAAGGGTGTGATCCAGACAGGTGTGAATACCAGCAAGACGGTACTGACAGGCGTAAAGAACACTGTCTGCTCTGGGGTTACCGGAGCTATGAACGTCGCAAAAGGCGTGATTCAGACTGGGGTGAACACCTCCAAAACCGTTCTCACAGGAGTGAAGAACACCGTTTGTTCCGGCGTTACAGGAGCGATGAATGTGGCCAAAGGGGTCATCCAAACCGGGGTCAACACCTCTAAGACGGTGCTGACTGGCGTGAAGAATACGGTGTGTAGTGGCGTTACTGGGGCTATGAATGTCGCCAAAGGCGTCATACAGACTGGTGTGAATACAaaggatcc
Plin4-charge swap	4xM3kd	SKDKLTGTVTTVGNCVTGAMSVATDKIQTGVGTSKDKLTGTVTTVGNCVTGAMSVATDKIQTGVGTSKDKLTGTVTTVGNCVTGAMSVATDKIQTGVGTSKDKLTGTVTTVGNCVTGAMSVATDKIQTGVGTKD	CTCGAGa**tgTCTAAAGACAAACTGACC**GGTACAGTGACGACTGTCGGGAATTGCGTGACTGGAGCAATGTCCGTTGCTACCGACAAGATCCAGACAGGGGTTGGGACGAGCAAAGATAAGCTTACCGGCACTGTGACTACAGTGGGCAATTGCGTTACCGGAGCCATGAGTGTCGCAACGGACAAGATACAGACTGGAGTGGGCACAAGCAAGGATAAACTGACGGGCACCGTTACCACCGTCGGAAACTGTGTCACCGGTGCGATGTCAGTGGCCACAGACAAGATCCAAACTGGCGTAGGGACTTCCAAGGATAAACTCACAGGGACCGTCACAACAGTGGGCAACTGTGTGACAGGTGCCATGTCTGTGGCTACCGATAAGATTCAGACTGGAGTAGGCACCaaGGATCC
Plin4-ch.swap,2T>V	P4-M7kt	SKdkLTGvvtTVgncVTGAMsVAvdkIQTGVgTSKdkLTGvvtTVgncVTGAMsVAvdkIQTGVgTSKdkLTGvvtTVgncVTGAMsVAvdkIQTGVgTSKdkLTGvvtTVgncVTGAMsVAvdkIQTGVgT	CTCGAGatgAGCAAAGACAAGCTTACCGGAGTCGTGACAACGGTCGGCAATTGCGTGACCGGTGCCATGTCAGTGGCTGTCGACAAGATACAGACAGGAGTGGGGACATCCAAGGATAAACTGACAGGGGTAGTGACCACAGTGGGCAACTGTGTGACTGGCGCAATGTCTGTGGCCGTTGACAAAATCCAGACTGGAGTAGGGACTAGCAAAGACAAACTGACTGGGGTTGTCACGACAGTGGGTAACTGTGTGACCGGAGCTATGAGTGTTGCCGTTGATAAGATCCAAACCGGCGTAGGGACCTCTAAGGATAAGCTCACCGGCGTTGTCACCACTGTGGGCAATTGCGTCACAGGTGCGATGTCCGTGGCAGTCGATAAGATTCAGACGGGAGTGGGCACTaaGGATCC

Supplementary References

1. Borhani, D. W., Rogers, D. P., Engler, J. A. & Brouillette, C. G. Crystal structure of truncated human apolipoprotein A-I suggests a lipid-bound conformation. *Proc Natl Acad Sci USA* **94,** 12291–12296 (1997).
2. Gautier, R., Douguet, D., Antonny, B. & Drin, G. HELIQUEST: a web server to screen sequences with specific -helical properties. *Bioinformatics* **24,** 2101–2102 (2008).
3. Krahmer, N. *et al.* Phosphatidylcholine Synthesis for Lipid Droplet Expansion Is Mediated by Localized Activation of CTP:Phosphocholine Cytidylyltransferase. *Cell Metab* **14,** 504–515 (2011).
4. Hristova, K. *et al.* An amphipathic alpha-helix at a membrane interface: a structural study using a novel X-ray diffraction method. *J Mol Biol* **290,** 99–117 (1999).
5. Creighton, T. E. Proteins. (*Macmillan*, 1993).
6. Hsieh, K. *et al.* Perilipin family members preferentially sequester to either triacylglycerol-specific or cholesteryl-ester-specific intracellular lipid storage droplets. *J Cell Sci* **125,** 4067–4076 (2012).
